# Analytic formulation of elastic field around edge dislocation adjacent to slanted free surface

**DOI:** 10.1098/rsos.220151

**Published:** 2022-06-08

**Authors:** Hiroyuki Shima, Yoshitaka Umeno, Takashi Sumigawa

**Affiliations:** ^1^ Department of Environmental Sciences, University of Yamanashi, 4-4-37, Takeda, Kofu, Yamanashi 400-8510, Japan; ^2^ Institute of Industrial Science, The University of Tokyo, Komaba, Meguro-ku, Tokyo 153-8505, Japan; ^3^ Department of Energy Conversion Science, Graduate School of Energy Science, Kyoto University, Sakyo-ku, Kyoto 606-8501, Japan

**Keywords:** dislocation theory, stress field, image force, traction-free boundary, surface effect

## Abstract

Explicit and tractable formulation of the internal stress field around edge dislocations is indispensable for considering the mechanics of fine crystalline solids, because the motion of edge dislocations in a slanted direction with respect to the free surface often plays a vital role in the plastic deformation of the solids under loading. In this study, we formulated an analytical solution for the stress distribution that occurs around edge dislocations embedded in a semi-infinite elastic medium. This formulation is based on the image force method and the Airy stress function method; it describes the variation in the stress distribution with changes in the slanted angle between the traction-free flat surface of the medium and the Burgers vector of the edge dislocation. Furthermore, our analytical solution shows that the attractive force acting on the edge dislocation due to the presence of the free surface is always perpendicular to the surface, regardless of the relative angle of the Burgers vector with the surface.

## 1. Introduction

A dislocation is a long linear defect in crystalline solids, originating from an abrupt local change in the arrangement of atoms. Dislocations are of two types: edge and screw. An edge dislocation is formed when a single extra monoatomic half-plane is inserted midway through the complete crystalline lattice of a metal or alloy, which distorts the nearby planes of atoms. This extra monoatomic surface imposes a substantial lattice strain and a resulting stress around the centre of the dislocation, thus significantly altering the elastic properties near this region. In addition, the presence of dislocations strongly influences the plasticity of crystals [1]; in fact, plastic deformation of crystals is caused by shear deformation, in which dislocations move in a specific slip direction along a specific slip plane.

The elasto-plastic mechanics of crystals with dislocations have been extensively researched, and many findings have been accumulated regarding the physical phenomena that occur on a macro scale [2–4]. Meanwhile, with the remarkable progress in microfabrication technology, the behaviour of dislocations in microscale crystalline samples has attracted renewed attention [5]. One of the physical effects that becomes more pronounced with decreasing sample size is the attractive force exerted by the free surface on the dislocations near the surface. This attractive force is attributed to the fact that as the dislocations move closer to the surface, less elastic energy is generated by the dislocations. In addition to the attraction force, the torque acting on dislocations due to the free surface has also been discussed [6,7]. Moreover, other interesting consequences of the surface effects have also been suggested for elastic media with voids [8,9] or cracks [2,10,11], multilayered media [12], and the interplay between the elasticity and dynamic motion of dislocations [13,14]. For micro-sized materials, the ratio of surface area to sample volume is so high that the aforementioned free-surface-driven phenomena are expected to be more prominent than those observed in macroscale samples [15,16].

**Figure 1.**
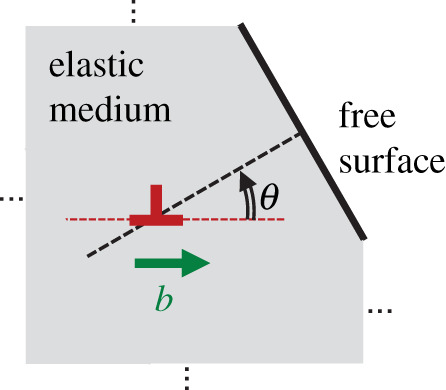
Edge dislocation inside a semi-infinite elastic medium. The dislocation is assumed to be near the flat free surface of the medium whose perpendicular is slanted relative to the Burgers vector by an angle *θ*.

To theoretically derive the attractive force, the image force method [2–4] is often used, which is a mechanical analogue to the image charge construction employed in electromagnetism [17]. In the image force method, the attraction force is computed as if it were due to an image dislocation, equidistant from the free surface but outside the material, and of the same magnitude as the original dislocation, but of the opposite sign. Using this method, the attractive force on the screw dislocations can be derived relatively easily, even for complex material configurations [18]. However, the counterpart problems regarding edge dislocations require a considerably lengthy mathematical derivation [19,20]. Therefore, most existing studies only describe cases in which the free surface is perpendicular to the Burgers vector of edge dislocations. Similar difficulties arise in the analysis of stress fields around edge dislocations. Calculation of the spatial distribution of the stress field created by dislocations is indispensable for estimating the physical properties of crystalline solids. Nevertheless, it is known that the image force method alone is not sufficient to accurately derive a stress field that satisfies a given boundary condition [21,22]; more complicated calculations are needed. This would be partly a reason why few detailed derivations of the stress field created by near-surface edge dislocations are thus far available for the case where the relative orientation between the free surface and the Burgers vector is arbitrary [19,23,24] (not limited to vertical or parallel) as shown in figure 1. Moreover, in actual metals, the edge dislocations that move in a slanted direction with respect to the free surface play a vital role in the plastic deformation [1]. Therefore, mathematical derivation of an explicit and tractable formula for the stress field of the system shown in figure 1 is important for inferring the surface effect on the fundamental properties of the edge dislocations near the free surface.

Against this backdrop, we present an explicit derivation of the stress field produced by an edge dislocation near a slanted free surface and the attractive force exerted on the edge dislocation. The present formulation is based on the image force method and the Airy stress function method, describing how the stress distribution varies with changes in the slanted angle between the free surface and the Burgers vector. The obtained solution shows that the attractive force acting on the edge dislocation is always perpendicular to the surface, regardless of the relative angle between the Burgers vector and the surface. It should be mentioned that the similar problem was addressed by Head [19] in the past; however, both the results we obtained and the method we used are different from those given earlier, as detailed at the end of §2 in the present article.

## 2. Formulation

### 2.1. Stress field due to real and imaginary edge dislocations

**Figure 2.**
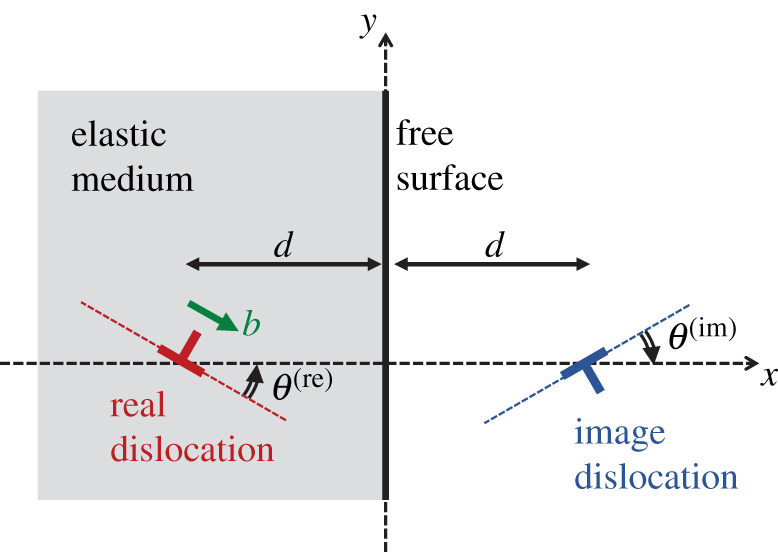
Illustration of the image force method. A negative dislocation with tilt angle $\theta^{\left(\mathrm{im}\right)}$ is virtually introduced at the opposite side of the free surface with respect to the positive dislocation tilted by $\theta^{\left(\mathrm{re}\right)}$ that exists inside the elastic medium. The distance *d* from the free surface to the dislocations is the same for both the real (left) and image (right) dislocations.

We consider an edge dislocation near the flat traction-free surface of an isotropic semi-infinite elastic medium. Figure 2 illustrates the configuration of the system embedded in the right-handed Cartesian coordinate system; the *z*-axis extends from the back to the front, perpendicular to the plane of the paper. The region of *x* ≤ 0 (shaded in figure 2) is occupied by the semi-infinite elastic medium, and the *y*–*z* plane serves as the free surface boundary to the motion of the dislocation. The dislocation is straight and parallel to the *z*-axis, positioned at (*x*, *y*) = (−*d*, 0), and is at a distance *d* from the free surface. As shown in the left side of figure 2, the Burgers vector of the edge dislocation (labelled by ‘real dislocation’ in the panel) is assumed to be tilted by an angle $\theta^{\left(\mathrm{re}\right)}$ with respect to the *x*-axis; the superscript ‘re’ denotes a real dislocation that exists within the actual elastic medium.

If the elastic medium were infinitely large in the three-dimensional space, this edge dislocation would produce a stress field over the entire space. The resulting stress components, denoted by $\sigma_{xx}^{\left(\mathrm{re}\right)}(x,y)$, $\sigma_{yy}^{\left(\mathrm{re}\right)}(x,y)$ and $\tau_{xy}^{\left(\mathrm{re}\right)}(x,y)$, can be derived using coordinate transformation (see appendices A and B). We can prove that all three components obey the following unique function:

2.1 $$\frac{G(\alpha_{c}\cos\theta^{\left(\mathrm{re}\right)}+\alpha_{s}\sin\theta^{\left(\mathrm{re}\right)})}{[{\left(x+d\right)}^{2}+y^{2}{]}^{2}},$$

with different definitions of *α*_*c*_ and *α*_*s*_. In equation (2.1), G is a material-dependent constant defined by

2.2 $$G=\frac{\mu b}{2\pi (1-\nu )},$$

where *μ* is the elastic shear modulus, *b* is the amplitude of the Burgers vector, and *ν* is Poisson’s ratio of the material under consideration. The definitions of *α*_*c*_ and *α*_*s*_ in equations (2.1) are as follows: In the case of $\sigma_{xx}^{\left(\mathrm{re}\right)}(x,y)$, we have

2.3 $$\begin{array}{rl} & \alpha_{c}=-3{\left(x+d\right)}^{2}y-y^{3}\\ \;\mathrm{and}\; & \alpha_{s}=-{\left(x+d\right)}^{3}+(x+d)y^{2}. \end{array}\}$$

For $\sigma_{yy}^{\left(\mathrm{re}\right)}(x,y)$,

2.4 $$\begin{array}{rl} & \alpha_{c}={\left(x+d\right)}^{2}y-y^{3}\\ \;\mathrm{and}\; & \alpha_{s}=-{\left(x+d\right)}^{3}-3(x+d)y^{2}. \end{array}\}$$

For $\tau_{xy}^{\left(\mathrm{re}\right)}(x,y)$, we have

2.5 $$\begin{array}{rl} & \alpha_{c}={\left(x+d\right)}^{3}-(x+d)y^{2}\\ \;\mathrm{and}\; & \alpha_{s}=-{\left(x+d\right)}^{2}y+y^{3}. \end{array}\}$$

Nevertheless, the stress field given by equation (2.1) will not be realized within the semi-infinite system, because the components $\sigma_{xx}^{\left(\mathrm{re}\right)}$ and $\tau_{xy}^{\left(\mathrm{re}\right)}$ do not vanish at *x* = 0. In other words, the stress field realized in the present system is modified to satisfy the condition that the flat surface at *x* = 0 should be free from any traction forces. The image force method is a theoretical approach for determining the stress field in a semi-infinite system by superposing the stress field produced by a virtual additional dislocation with a reversed sign onto the stress field produced by the real dislocation within the medium. In the present system, this is partly achieved by virtually introducing an image edge dislocation with a negative sign at (*x*, *y*) = (*d*, 0), as shown in the right side of figure 2. The tilt angle of this image dislocation is $\theta^{\left(\mathrm{im}\right)}$. If the image dislocation is solely present at (*x*, *y*) = (*d*, 0) in an infinitely large elastic medium, it produces a stress field denoted by $\sigma_{xx}^{\left(\mathrm{im}\right)}(x,y)$, $\sigma_{yy}^{\left(\mathrm{im}\right)}(x,y)$ and $\tau_{xy}^{\left(\mathrm{im}\right)}(x,y)$, which are also expressed in equation (2.1); however, the following three modifications are required:

2.6 $$G\to -G,\;\theta^{\left(\mathrm{re}\right)}\to \theta^{\left(\mathrm{im}\right)},\;x+d\to x-d.$$

In fact, if we fix the tilt angles of the two dislocations as

2.7 $$\theta^{\left(\mathrm{re}\right)}=\theta \;\text{and}\;\theta^{\left(\mathrm{im}\right)}=-\theta ,$$

then the sum of the normal stress components becomes zero at every point on the line *x* = 0:

2.8 $$\sigma_{xx}^{\left(\mathrm{re}\right)}(0,y)+\sigma_{xx}^{\left(\mathrm{im}\right)}(0,y)=0$$

and

2.9 $$\sigma_{yy}^{\left(\mathrm{re}\right)}(0,y)+\sigma_{yy}^{\left(\mathrm{im}\right)}(0,y)=0,$$

at an arbitrary *y*. Equation (2.8) ensures that the traction-free surface condition is partly achieved, by virtually introducing the image dislocation at (*d*, 0).

Meanwhile, the sum of shear components does not yet become zero along *x* = 0; hence,

2.10 $$\tau_{xy}^{\left(\mathrm{re}\right)}(0,y)+\tau_{xy}^{\left(\mathrm{im}\right)}(0,y)=2G\frac{\left(y^{2}-d^{2})(y\sin\theta -d\cos\theta \right)}{{\left(y^{2}+d^{2}\right)}^{2}}.$$

This indicates that an additional stress field should be superimposed to cancel the component expressed by equation (2.10). This additional stress field can be identified using a method based on Airy’s stress function, as explained in the next section.

### 2.2. Airy’s stress function approach

The linear elasticity theory states that any in-plane strain problem in two dimensions can be reduced to a partial differential equation with a single unknown, *ϕ*(*x*, *y*) [25]:

2.11 $$\frac{\partial^{4}\phi }{\partial x^{4}}+2\frac{\partial^{4}\phi }{\partial x^{2}\partial y^{2}}+\frac{\partial^{4}\phi }{\partial y^{4}}=0.$$

Once the solution of *ϕ* in a particular domain of interest is obtained under the given boundary conditions, the stress components within the domain can be derived through partial differentiation of *ϕ*(*x*, *y*) (see equation (2.12)).

Thus, our immediate task is to derive such a solution of equation (2.11), denoted by $\phi^{\left(\mathrm{ex}\right)}(x,y)$, that cancels the shear components given by equation (2.10); the three components of the stress field derived from the solution are

2.12 $$\sigma_{xx}^{\left(\mathrm{ex}\right)}=\frac{\partial^{2}\phi^{\left(\mathrm{ex}\right)}}{\partial y^{2}},\;\sigma_{yy}^{\left(\mathrm{ex}\right)}=\frac{\partial^{2}\phi^{\left(\mathrm{ex}\right)}}{\partial x^{2}},\;\tau_{xy}^{\left(\mathrm{ex}\right)}=\frac{-\partial^{2}\phi^{\left(\mathrm{ex}\right)}}{\partial x\partial y}.$$

Accordingly, each component of the actual stress field generated within the present semi-infinite system can be expressed as the sum of three components (real, image and excess), as follows:

2.13 $$\sigma_{xx}(x,y)=\sum_{j}\sigma_{xx}^{\left(j\right)}(x,y),$$

2.14 $$\sigma_{yy}(x,y)=\sum_{j}\sigma_{yy}^{\left(j\right)}(x,y)$$

2.15 $$\;\mathrm{and}\;\tau_{xy}(x,y)=\sum_{j}\tau_{xy}^{\left(j\right)}(x,y),$$

with j = re, im, ex. Furthermore, since the boundary condition at the free surface requires *σ*_*xx*_(*x*, *y*) and *τ*_*xy*_(*x*, *y*) to vanish at *x* = 0, the two components $\sigma_{xx}^{\left(\mathrm{ex}\right)}(x,y)$ and $\tau_{xy}^{\left(\mathrm{ex}\right)}(x,y)$, derived from the solution $\phi^{\left(\mathrm{ex}\right)}(x,y)$ must satisfy the following relations:

2.16 $$\sigma_{xx}^{\left(\mathrm{ex}\right)}(0,y)=0$$

and

2.17 $$\tau_{xy}^{\left(\mathrm{ex}\right)}(0,y)=-2G\frac{\left(y^{2}-d^{2})(y\sin\theta -d\cos\theta \right)}{{\left(y^{2}+d^{2}\right)}^{2}}.$$

We hypothesize that such a solution, $\phi^{\left(\mathrm{ex}\right)}(x,y)$, that satisfies both equations (2.16) and (2.17) can be obtained using variable separation:

2.18 $$\phi^{\left(\mathrm{ex}\right)}(x,y)=\xi (x)\eta (y).$$

Substituting it to equation (2.11), we have

2.19 $$\frac{\partial^{4}\xi }{\partial x^{4}}+\frac{2}{\eta }\frac{\partial^{2}\xi }{\partial x^{2}}\frac{\partial^{2}\eta }{\partial y^{2}}+\frac{\xi }{\eta }\frac{\partial^{4}\eta }{\partial y^{4}}=0,$$

which implies that the two terms,

2.20 $$\frac{2}{\eta }\frac{\partial^{2}\eta }{\partial y^{2}}\;\mathrm{and}\;\frac{1}{\eta }\frac{\partial^{4}\eta }{\partial y^{4}},$$

are constants (i.e. independent of *y*). If we set the first term in equation (2.20) to be equal to the constant −2*k*^2^ with *k* > 0, *η*(*y*) becomes

2.21 $$\eta (y)=c_{1}\sin ky+c_{2}\cos ky,$$

with appropriate constants *c*_1_ and *c*_2_. Substituting equation (2.21) into (2.19), we have

2.22 $$\frac{\partial^{4}\xi }{\partial x^{4}}-2k^{2}\frac{\partial^{2}\xi }{\partial x^{2}}+k^{4}\xi =0,$$

whose solutions are

2.23 $$\xi (x)=(c_{3}+c_{4}x)e^{kx}+(c_{5}+c_{6}x)\;e^{-kx}\;(k>0).$$

Because the stress field produced within the elastic medium far from the free surface should converge to zero, *ξ*(*x*) must vanish at the limit of *x* → −∞, which implies that *c*_5_ = *c*_6_ = 0. In addition, because the traction-free condition at the surface, $\sigma_{xx}^{\left(\mathrm{ex}\right)}(0,y)=0$, is satisfied only if *ξ*(*x*)∂^2^*η*(*y*)/∂*y*^2^ = 0 at *x* = 0, *ξ*(*x*) mush vanish at *x* = 0, which implies that *c*_3_ = 0. As a consequence, the solution of $\phi^{\left(\mathrm{ex}\right)}(x,y)$ that satisfies the boundary conditions reads

2.24 $$\phi^{\left(\mathrm{ex}\right)}(x,y)=xe^{kx}(a_{1}\sin ky+a_{2}\cos ky),$$

where *a*_1_ = *c*_1_ *c*_4_ and *a*_2_ = *c*_2_ *c*_4_.

Note that equation (2.24) is a solution for a specific value of the positive constant *k*, while the value of *k* can be arbitrarily chosen. The appropriate values of *a*_1_ and *a*_2_ are also dependent on *k*. From the principle of superposition, therefore, it follows that the general solution of $\phi^{\left(\mathrm{ex}\right)}(x,y)$ that satisfies $\sigma_{xx}^{\left(\mathrm{ex}\right)}(0,y)=0$ is a linear combination of all the solutions corresponding to different *k* values as given by

2.25 $$\phi^{\left(\mathrm{ex}\right)}(x,y)=\int_{0}^{\infty }a_{1}(k)x\;e^{kx}\sin ky\;dk+\int_{0}^{\infty }a_{2}(k)x\;e^{kx}\cos ky\;dk,$$

where we explicitly expressed that *a*_1_ and *a*_2_ depend on *k*. Partial differentiations of it according to equation (2.12) yield the stress components associated with $\phi^{\left(\mathrm{ex}\right)}(x,y)$:

2.26 $$\sigma_{xx}^{\left(\mathrm{ex}\right)}(x,y)=I_{s,1}(-kx;x,y)+I_{c,2}(-kx;x,y),$$

2.27 $$\sigma_{yy}^{\left(\mathrm{ex}\right)}(x,y)=I_{s,1}(2+kx;x,y)+I_{c,2}(2+kx;x,y)$$

2.28 $$\;\mathrm{and}\;\tau_{xy}^{\left(\mathrm{ex}\right)}(x,y)=I_{s,2}(1+kx;x,y)+I_{c,1}(-1-kx;x,y),$$

where

2.29 $$I_{s,i}(u;x,y)=\int_{0}^{\infty }u\;ka_{i}(k)\;e^{kx}\sin ky\;dk$$

and

2.30 $$I_{c,i}(u;x,y)=\int_{0}^{\infty }u\;ka_{i}(k)\;e^{kx}\cos ky\;dk,$$

with *i* = 1 or 2.

The remaining task is to find the *k*-dependences of *a*_1_(*k*) and *a*_2_(*k*) that suffice for $\tau_{xy}^{\left(\mathrm{ex}\right)}(0,y)$ to cancel the shear-stress contribution of equation (2.10) from the real and image dislocations. From equations (2.17) and (2.28), this requirement regarding $\tau_{xy}^{\left(\mathrm{ex}\right)}(0,y)$ is expressed as

2.31 $$I_{s,2}(1;0,y)+I_{c,1}(-1;0,y)=\frac{-2G(y^{2}-d^{2})(y\sin\theta -d\cos\theta )}{{\left(y^{2}+d^{2}\right)}^{2}}.$$

Applying the inverse cosine and sine Fourier transforms to both sides of equation (2.31), we obtain

2.32 $$-ka_{1}(k)=\frac{-2G}{\pi }\int_{-\infty }^{\infty }\frac{\left(y^{2}-d^{2})(y\sin\theta -d\cos\theta \right)}{{\left(y^{2}+d^{2}\right)}^{2}}\cos ky\;dy$$

and

2.33 $$ka_{2}(k)=\frac{-2G}{\pi }\int_{-\infty }^{\infty }\frac{\left(y^{2}-d^{2})(y\sin\theta -d\cos\theta \right)}{{\left(y^{2}+d^{2}\right)}^{2}}\sin ky\;dy.$$

The integrations in equations (2.32) and (2.33) can be performed based on the residue theorem [26] (see appendix C), which yields

2.34 $$ka_{1}(k)=2G\;kd\;e^{-kd}\cos\theta$$

and

2.35 $$ka_{2}(k)=2G\;(kd-1)\;e^{-kd}\sin\theta .$$

Substitution of these into the integrands of equations (2.29) and (2.30), followed by substitution of the integration results into the formulas of equations (2.26)–(2.28), allow us to compute the suitable stress components, $\sigma_{xx}^{\left(\mathrm{ex}\right)}$, $\sigma_{yy}^{\left(\mathrm{ex}\right)}$, $\tau_{xy}^{\left(\mathrm{ex}\right)}$, derived from $\phi^{\left(\mathrm{ex}\right)}$ that satisfy the free-surface conditions of *σ*_*xx*_(0, *y*) = 0 and *τ*_*xy*_(0, *y*) = 0. Explicit forms of the stress components in terms of *x* and *y* are

2.36 $$\frac{2G(\gamma_{c}\cos\theta +\gamma_{s}\sin\theta )}{{\left[{\left(x-d\right)}^{2}+y^{2}\right]}^{3}},$$

with *γ*_*c*_ and *γ*_*s*_ defined as below: in the case of $\sigma_{xx}^{\left(\mathrm{ex}\right)}(x,y)$, we have

2.37 $$\begin{array}{rl} & \gamma_{c}=-6dx{\left(x-d\right)}^{2}y+2dxy^{3}\\ \;\mathrm{and}\; & \gamma_{s}=x(x+d){\left(x-d\right)}^{3}-6dx(x-d)y^{2}-xy^{4}. \end{array}\}$$

For $\sigma_{yy}^{\left(\mathrm{ex}\right)}(x,y)$,

2.38 $$\begin{array}{rl} & \gamma_{c}=2d(x+2d){\left(x-d\right)}^{2}y-2d(3x-2d)y^{3}\\ \;\mathrm{and}\; & \gamma_{s}=x(x-3d){\left(x-d\right)}^{3}\\ & \;+2(x-d)(2x^{2}-dx+2d^{2})y^{2}+(3x-4d)y^{4}. \end{array}\}$$

Finally, for $\tau_{xy}^{\left(\mathrm{ex}\right)}(x,y)$,

2.39 $$\begin{array}{cc} & \gamma_{c}=d(x+d){\left(x-d\right)}^{3}-6\;dx(x-d)y^{2}+dy^{4},\\ \;\mathrm{and}\; & \gamma_{s}={\left(x-d\right)}^{2}(x^{2}+4\;dx+d^{2})y-2\;dxy^{3}-y^{5}. \end{array}\}$$

It is readily evident that the components $\sigma_{xx}^{\left(\mathrm{ex}\right)}(x,y)$ and $\tau_{xy}^{\left(\mathrm{ex}\right)}(x,y)$ satisfy the boundary conditions given in equations (2.16) and (2.17), respectively.

The complete stress distribution within the semi-infinite medium is determined by superposing the stress fields of the real edge dislocation at (−*d*, 0) and the image dislocation at (*d*, 0), and the stress field derived from Airy’s stress function $\phi^{\left(\mathrm{ex}\right)}(x,y)$, as shown in equations (2.13)–(2.15).

As briefly mentioned in the Introduction, we are aware that the stress components under similar conditions to those dealt with in the present work have been considered earlier by Head [19] using a different approach. However, Head’s solution does not satisfy the equilibrium conditions expressed by

2.40 $$\frac{\partial \sigma_{xx}}{\partial x}+\frac{\partial \tau_{xy}}{\partial y}=0\;\text{and}\;\frac{\partial \tau_{xy}}{\partial x}+\frac{\partial \sigma_{yy}}{\partial y}=0.$$

The conditions of equation (2.40) ensure that the forces acting on the inside of the system are balanced with each other and thus the system is stationary. We believe, therefore, that it remains to be debated whether the Head’s solution that does not satisfy the equilibrium condition is consistent with the stress field in actual elastic media. On the other hand, that our solutions of the stress components satisfy the equilibrium condition can be proved straightforwardly by substituting them into equation (2.40).

## 3. Results

### 3.1. Total stress distribution

**Figure 3.**
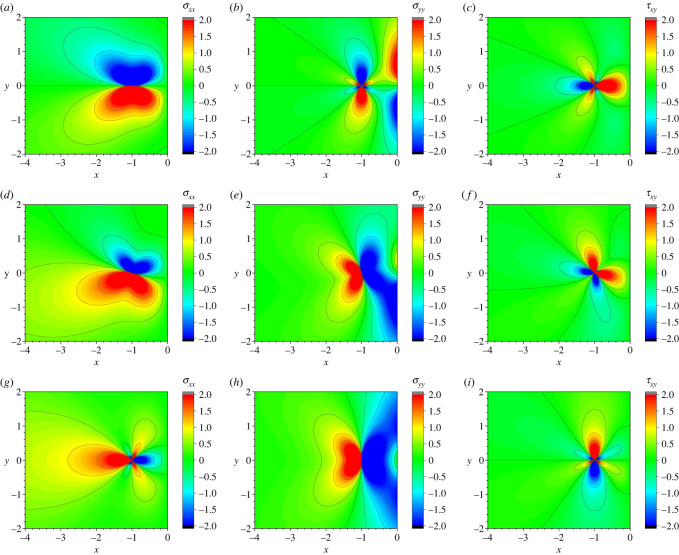
Spatial distributions of the three stress components *σ*_*xx*_, *σ*_*yy*_, *τ*_*xy*_ produced by an edge dislocation at (*x*, *y*) = ( − 1, 0), whose direction is parallel to the free surface at *x* = 0. The tilt angle of the Burgers vector in the surface normal direction (i.e. the *x*-axis direction) is set as (*a*)–(*c*) *θ* = 0, (*d*)–(*f* ) *θ* = *π*/4, (*g*)–(*i*) *θ* = *π*/2. In all the plots, the upper and lower limits of the contour lines are 2.0 and −2.0, respectively, with G/b and *b* being the unit of stress and the length scale, respectively.

Figure 3 shows the spatial distributions of the three stress components produced by the real edge dislocation at (*x*, *y*) = (−1, 0). The tilt angle *θ* of the Burgers vector with the *x*-axis is *θ* = 0 in (*a*)–(*c*), *θ* = *π*/4 in (*d*)–(*f*), and *θ* = *π*/2 in (*g*)–(*i*). In all the plots, G/b and *b* are taken as the unit of stress and the length scale, respectively. In many cases, *μ* of a metal reaches in the order of tens of gigapascals, and *b* is a few angstroms. Assuming Poisson ratio *ν* to be 0.3, therefore, G/b is estimated to be several gigapascals.

All the plots show a clear deviation in the stress field from the field generated in an infinitely large system with no surface boundary; in the latter, a vertical or lateral symmetry around the core of dislocation should be observed based on the function form given by equations (A 3)–(A 5). In particular, the *σ*_*yy*_ distribution close to the free surface exhibits a significant deviation from the symmetric distribution observed in an infinite system; the figure shows that because of the region having a non-zero stress component expanding significantly toward the edge of the sample, a large tensile or compressive force along the *y*-direction acts on the free surface. The region that deviates considerably from the stress distribution in such an infinite system extends from the centre of the dislocation to a point several times the magnitude of the Burgers vector.

Notably, the physical quantity measured in an actual experiment is often strain rather than stress. By using the analytical solution for the stress distribution derived herein, the strain distribution can be easily obtained from the relational expression below:

3.1 $$\varepsilon_{xx}(x,y)=E^{-1}[(1-\nu^{2})\sigma_{xx}-\nu (1+\nu )\sigma_{yy}],$$

3.2 $$\varepsilon_{yy}(x,y)=E^{-1}[(1-\nu^{2})\sigma_{yy}-\nu (1+\nu )\sigma_{xx}]$$

3.3 $$\;\mathrm{and}\;\varepsilon_{xy}(x,y)={\left(2\mu \right)}^{-1}\tau_{xy},$$

where *E* is Young’s modulus of the material considered.

### 3.2. Peach–Koehler force

We now consider the Peach–Koehler force exerted on the real-edge dislocation at (−*d*, 0). The *x*-and *y*-components of the force, *f*_*x*_ and *f*_*y*_, can be written as [27,28]

3.4 $$f_{x}=b\tau_{xy}^{*}\cos\theta -b\sigma_{yy}^{*}\sin\theta$$

and

3.5 $$f_{y}=-b\sigma_{xx}^{*}\cos\theta +b\tau_{xy}^{*}\sin\theta .$$

as proven in appendix D. Here, the abbreviated notation $\sigma_{xx}^{*}$ indicates that

3.6 $$\sigma_{xx}^{*}=\sigma_{xx}^{\left(\mathrm{im}\right)}(-d,0)+\sigma_{ij}^{\left(\mathrm{ex}\right)}(-d,0),$$

and the same applies to both $\sigma_{yy}^{*}$ and $\tau_{xy}^{*}$.

It follows from the formulation given in §2 that the values of the image dislocation-derived stress components at (*x*, *y*) = (−*d*, 0), are

3.7 $$\sigma_{xx}^{\left(\mathrm{im}\right)}(-d,0)=\sigma_{yy}^{\left(\mathrm{im}\right)}(-d,0)=\frac{G}{2d}\sin\theta \;\text{and}\;\tau_{xy}^{\left(\mathrm{im}\right)}(-d,0)=\frac{G}{2d}\cos\theta .$$

Regarding the $\phi^{\left(\mathrm{ex}\right)}$-derived components, we have

3.8 $$\sigma_{xx}^{\left(\mathrm{ex}\right)}(-d,0)=0,\;\sigma_{yy}^{\left(\mathrm{ex}\right)}(-d,0)=\frac{-G}{d}\sin\theta \;\text{and}\;\tau_{xy}^{\left(\mathrm{ex}\right)}(-d,0)=0.$$

Substituting these values into equations (3.4) and (3.5), we obtain a highly concise expression:

3.9 $$f_{x}=\frac{Gb}{2d}\;\mathrm{and}\;f_{y}=0.$$

Since the sign of *f*_*x*_ is always positive, the force acting on the edge dislocation is always directed toward the free surface. Notably, when considering the motion of edge dislocations, the component of the force acting on the dislocations that is parallel to the slip plane is often important. In such a case, it is necessary to only decompose the attraction force into parallel (*f*_glide_) and normal (*f*_climb_) components in the gliding direction (i.e. ***b***); then, we have *f*_glide_ = *f*_*x*_ cos *θ* and *f*_climb_ = *f*_*x*_ sin *θ*. In actual crystalline samples, edge dislocations start to move along the slip plane only when the force *f*_glide_ exceeds various frictional forces.

An important implication of the concise expression, equation (3.9), is that the force exerted on the edge dislocation near the free surface is always oriented normal to the surface, as consistent with the earlier theoretical work [29,30]. This observation may seem to be in contrast to our finding that the stress distribution created by the edge dislocation changes differently depending on the change in the direction of the Burgers vector, while the same conclusion can be obtained by an energetics argument on the near-surface edge dislocation [11]. It should also be mentioned that under certain conditions, the direction of the attraction force does not have to be perpendicular to the free surface if it is uneven rather than flat [31].

Another interesting finding is that the magnitude of the attraction force is independent of the tilt angle *θ* of the Burgers vector. It depends only on the distance *d* from the surface; specifically, it is inversely proportional to this distance. The *θ*-independence of the attraction force may also seem counterintuitive, given that the stress distribution is strongly dependent on *θ*, while it has been proved exactly through our formulation. Special attention should be paid to the fact that the magnitude of the action force, Gb/(2d), is identical to that of the force generated when two edge dislocations with Burgers vectors having opposite signs are located on a straight line parallel to the Burgers vector and separated by a distance of 2*d* (i.e. when $\theta^{\left(\mathrm{re}\right)}=\theta^{\left(\mathrm{im}\right)}=0$ in figure 2). That is, the attraction force from the free surface acting on the edge dislocations in the semi-infinite elastic medium is determined only by the contribution from the image dislocation; the stress distribution created by the Airy function offers no contribution. This phenomenon is known to occur in a system with *θ* = 0; however, we found that it also holds true for any choice of *θ*.

## 4. Summary

Herein, we derived an analytical solution for the stress field distribution around edge dislocations positioned near the slanted free surface of a semi-infinite elastic medium. The explicit function forms of the stress components were derived using the image force method and Airy’s function method. The findings revealed significant variations in the stress distribution in response to changes in the direction of the Burgers vector of the dislocation. By contrast, the stress field, however, the attraction force exerted by the free surface on the edge dislocation is independent of the direction of the Burgers vector; it follows a universal function that is inversely proportional to the distance between the dislocation and the free surface.

## Figures and Tables

**Figure 4.**
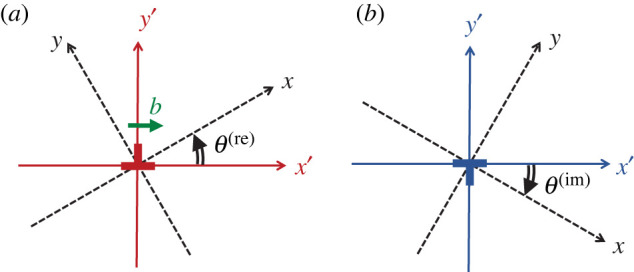
Schematics of the coordinate system rotation around the dislocation line (i.e. about the *z*-axis). The counterclockwise rotation is the positive direction. (*a*) Rotation of the *x*′–*y*′ system to the *x*–*y* system by an angle $\theta^{\left(\mathrm{re}\right)}(>0)$ around the real (positive-signed) edge dislocation. (*b*) Rotation by angle $\theta^{\left(\mathrm{im}\right)}(<0)$ around the image (negative-signed) edge dislocation.

**Figure 5.**
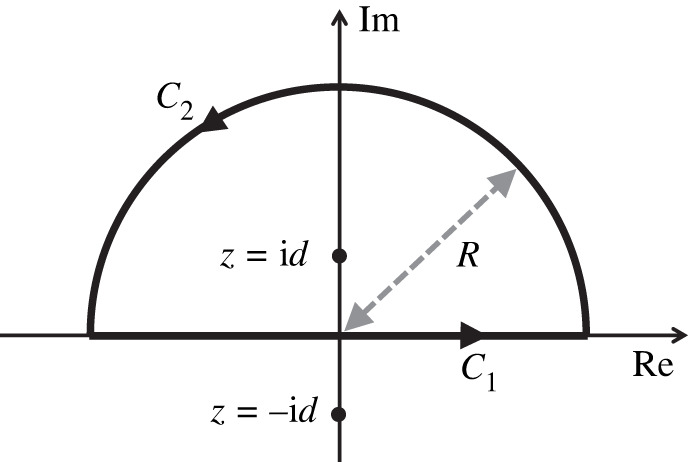
Semi-circular integral path on the complex plane, which allows us to calculate the integrals in equations (2.32) and (2.33). The integrands have two points of singularity, *z* = i*d* and *z* = −i*d*, among which only the former point is surrounded by the integral path.

## Data Availability

All data and models generated or used during the study appear in the submitted article.

## Appendix A. Stress field around-edge dislocation

Suppose that an infinitely long, straight-edge dislocation extends along the *z*′ axis in the *x*′–*y*′–*z*′ Cartesian coordinate system and that its core is located at the origin of the *x*′–*y*′ coordinate plane, as illustrated in figure 4*a*. The stress field generated by this edge dislocation can be computed using the Airy stress function [32]:

A 1 $$\phi (x^{\prime },y^{\prime })=-Gy^{\prime }\log\left(\sqrt{x^{\prime 2}+y^{\prime 2}}\right).$$

Here, G is a material-dependent constant defined by

A 2 $$G=\frac{\mu b}{2\pi (1-\nu )},$$

where *μ* is the elastic shear modulus, *b* is the amplitude of the Burgers vector, and *ν* is Poisson’s ratio of the material under consideration. The function *ϕ* in equation (A 1) is a solution to the partial differential equation shown in inequation (2.11). Among many possible solutions, this explicit functional form was identified by imposing physical constraints to the general solution involving arbitrary constants (see Lardner [33] for the detailed derivation).

When this Airy function of equation (A 1) is partially differentiated up to the second order with *x*, *y*, or both in the same manner as in equation (2.12), the stress distribution around the edge dislocation is expressed as follows:

A 3 $$\sigma_{x^{\prime }x^{\prime }}=G\;\frac{-y^{\prime }(3x^{\prime 2}+y^{\prime 2})}{{\left(x^{\prime 2}+y^{\prime 2}\right)}^{2}},$$

A 4 $$\sigma_{y^{\prime }y^{\prime }}=G\;\frac{y^{\prime }(x^{\prime 2}-y^{\prime 2})}{{\left(x^{\prime 2}+y^{\prime 2}\right)}^{2}}$$

A 5 $$\;\mathrm{and}\;\tau_{x^{\prime }y^{\prime }}=G\;\frac{x^{\prime }(x^{\prime 2}-y^{\prime 2})}{{\left(x^{\prime 2}+y^{\prime 2}\right)}^{2}}$$

and *σ*_*z*′*z*′_ = *ν*(*σ*_*x*′*x*′_ + *σ*_*y*′*y*′_), *τ*_*z*′*x*′_ = 0, *τ*_*y*′*z*′_ = 0. In the main text, the latter three components that act on the plane normal to the *z*-axis are not necessary to be taken into consideration, since we have assumed the in-plane strain problem in which the dislocation line is parallel to the free surface.

If the sign of the Burgers vector is reversed from positive to negative, then the stress components of the negative edge dislocation are obtained by replacing G with −G in the expressions above. Figure 4*b* shows the configuration of the negative edge dislocation in the *x*′–*y*′–*z*′ Cartesian coordinate system.

## Appendix B. Coordinate transformation

Consider a rotation of the *x*′–*y*′–*z*′ coordinate system about the *z*′-axis by an angle of *θ* (figure 4); the sign of *θ* is set to be positive for counterclockwise rotation, and negative for clockwise rotation. The coordinates after rotation (*x*, *y*) are related to the original coordinates, (*x*′, *y*′), by

B 1 $$x=x^{\prime }\cos\theta +y^{\prime }\sin\theta$$

and

B 2 $$y=-x^{\prime }\sin\theta +y^{\prime }\cos\theta .$$

In addition, the theory of elasticity states that the stress components in terms of the rotated coordinates are

B 3 $$\sigma_{xx}=\frac{1}{2}(\sigma_{x^{\prime }x^{\prime }}+\sigma_{y^{\prime }y^{\prime }})+\frac{1}{2}(\sigma_{x^{\prime }x^{\prime }}-\sigma_{y^{\prime }y^{\prime }})\cos2\theta +\tau_{x^{\prime }y^{\prime }}\sin2\theta ,$$

B 4 $$\sigma_{yy}=\frac{1}{2}(\sigma_{x^{\prime }x^{\prime }}+\sigma_{y^{\prime }y^{\prime }})-\frac{1}{2}(\sigma_{x^{\prime }x^{\prime }}-\sigma_{y^{\prime }y^{\prime }})\cos2\theta -\tau_{x^{\prime }y^{\prime }}\sin2\theta$$

B 5 $$\;\mathrm{and}\;\tau_{xy}=-\frac{1}{2}(\sigma_{x^{\prime }x^{\prime }}-\sigma_{y^{\prime }y^{\prime }})\sin2\theta +\tau_{x^{\prime }y^{\prime }}\cos2\theta .$$

Using the results presented in appendix A, as well as through coordinate translation based on −*d* (or +*d*) along the *x*-axis, we obtain the explicit *x*- and *y*-dependences of the stress components $\sigma_{xx}^{\left(\mathrm{re}\right)},\sigma_{yy}^{\left(\mathrm{re}\right)},\tau_{xy}^{\left(\mathrm{re}\right)}$ produced by the real dislocations and $\sigma_{xx}^{\left(\mathrm{im}\right)},\sigma_{yy}^{\left(\mathrm{im}\right)},\tau_{xy}^{\left(\mathrm{im}\right)}$ produced by the image dislocations, as discussed in the present analysis; see equations (2.1)–(2.6) in the main text for the explicit conclusions.

## Appendix C. Residue theorem

This appendix explains how to compute the integrations given by equations (2.32) and (2.33) for obtaining the coefficients *ka*_1_(*k*) and *ka*_2_(*k*) that are important for determining the stress components derived from the field $\phi^{\left(\mathrm{ex}\right)}(x,y)$. The computation is based on the residue theorem, which is a powerful tool for evaluating the line integrals of analytic functions over closed curves.

Assume a complex plane associated with the complex-valued variable *z* (figure 5). *C*_1_ denotes the line segment [−*R*, *R*] on the real axis (oriented to the right), and *C*_2_ represents the semicircular arc of radius *R* on the upper half-plane centred at the origin, in the counterclockwise direction. Connecting them, we obtain a closed curve, designated as *C*(= *C*_1_ + *C*_2_).

Next, we consider the contour integral $\oint_{C}f\;(z)dz$ with respect to the complex-valued function *f*(*z*) defined by

C 1 $$f\;(z)=\frac{\left(z^{2}-d^{2})(z\sin\theta -d\cos\theta \right)}{{\left(z^{2}+d^{2}\right)}^{2}}\;e^{ikz}\;(k>0).$$

It follows from equation (C 1) that *f*(*z*) has two singular points at *z* = i*d* and *z* = −i*d*, both of which are second order. Of these, only the former is included inside *C*. The residue theorem states that

C 2 $$\oint_{C}f\;(z)\;dz=2\pi i\;\mathrm{Res}(id),$$

where Res(*z*) denotes the residue of *f*(*z*) at the singular point at *z*. In the present case, we have

C 3 $$\begin{array}{rl} \mathrm{Res}(id) & ={\frac{d}{dz}\left[\frac{\left(z^{2}-d^{2})(z\sin\theta -d\cos\theta \right)}{{\left(z+id\right)}^{2}}\;e^{ikz}\right]|}_{z=id}\\ & =\frac{e^{-kd}}{2}[(1-kd)\sin\theta -ikd\cos\theta ]. \end{array}$$

In the limit of *R* → ∞, the contribution from *C*_2_ to the line integral $\oint_{C}f(z)dz$ vanishes; thus, we have

C 4 $$\int_{-\infty }^{\infty }\frac{\left(z^{2}-d^{2})(z\sin\theta -d\cos\theta \right)}{{\left(y^{2}+d^{2}\right)}^{2}}\;e^{iky}\;dy=\pi \;e^{-kd}[kd\cos\theta +i(1-kd)\sin\theta ].$$

Extracting the real and imaginary parts from both sides of equation (C 4) and then substituting them into equations (2.32) and (2.33), respectively, we obtain

C 5 $$ka_{1}(k)=2G\;kd\;e^{-kd}\cos\theta$$

and

C 6 $$ka_{2}(k)=2G\;(kd-1)\;e^{-kd}\sin\theta .$$

## Appendix D. Peach–Koehler force

Dislocations that exist in a stress field experience forces from that field. This force, called the Peach–Koehler force, is expressed by

D 1 $$f=bP\times \frac{ds}{ds},$$

where ***b*** is the Burgers vector of the dislocation, d***s*** is an infinitesimal vector in the direction parallel to the dislocation line, and ***P*** is the stress tensor written by a symmetric matrix of

D 2 $$P=\left(\begin{array}{ccc} \sigma_{xx} & \sigma_{xy} & \sigma_{xz}\\ \sigma_{yx} & \sigma_{yy} & \sigma_{yz}\\ \sigma_{zx} & \sigma_{zy} & \sigma_{zz} \end{array}\right).$$

A more intuitive expression of ***f*** can be obtained if we consider matrix ***P*** as a combination of three column vectors, such as

D 3 $$P=\left(\begin{array}{ccc} p^{\left(x\right)} & p^{\left(y\right)} & p^{\left(z\right)} \end{array}\right),$$

with

D 4 $$p^{\left(x\right)}=\sigma_{xx}i+\sigma_{yx}j+\sigma_{zx}k,$$

D 5 $$p^{\left(y\right)}=\sigma_{xy}i+\sigma_{yy}j+\sigma_{zy}k$$

D 6 $$\;\mathrm{and}\;p^{\left(z\right)}=\sigma_{xz}i+\sigma_{yz}j+\sigma_{zz}k,$$

where ***i***, ***j*** *and* ***k*** are unit vectors in the *x*-, *y*- and *z*-directions, respectively. Then, the vector ***b******P*** is expressed as

D 7 $$bP=(b\cdot p^{\left(x\right)})\;i+(b\cdot p^{\left(y\right)})\;j+(b\cdot p^{\left(z\right)})\;k.$$

This result allows us to evaluate the magnitude and direction of force ***f***, once the two vectors of ***b*** and d***s*** are specified.

In the main text, we considered the case in which ***b*** and d***s*** are expressed as

D 8 b=bcos⁡θi−bsin⁡θj,ds=ds k.

Thus, the Peach–Koehler force ***f*** = *f*_*x*_***i*** + *f*_*y*_***j*** is composed of the following components:

D 9 $$f_{x}=b\cdot p^{\left(y\right)}=b\sigma_{xy}\cos\theta -b\sigma_{yy}\sin\theta$$

and

D 10 $$f_{y}=-b\cdot p^{\left(x\right)}=-b\sigma_{xx}\cos\theta +b\sigma_{yx}\sin\theta .$$

Rewriting both *σ*_*xy*_ and *σ*_*yx*_ with *τ*_*xy*_, we obtain equations (3.4) and (3.5).
